# Cytotoxicity and *In Vitro* Antileishmanial Activity of Antimony (V), Bismuth (V), and Tin (IV) Complexes of Lapachol

**DOI:** 10.1155/2013/961783

**Published:** 2013-05-25

**Authors:** Marcele Neves Rocha, Paula Monalisa Nogueira, Cynthia Demicheli, Ludmila Gonçalvez de Oliveira, Meiriane Mariano da Silva, Frédéric Frézard, Maria Norma Melo, Rodrigo Pedro Soares

**Affiliations:** ^1^Centro de Pesquisas René Rachou, Fundação Oswaldo Cruz/FIOCRUZ, 30190-002 Belo Horizonte, MG, Brazil; ^2^Departamento de Química, Instituto de Ciências Exatas, Universidade Federal de Minas Gerais, 31270-901 Belo Horizonte, MG, Brazil; ^3^Departamento de Fisiologia e Biofísica, Instituto de Ciências Biológicas, Universidade Federal de Minas Gerais, 31270-901 Belo Horizonte, MG, Brazil; ^4^Departamento de Parasitologia, Instituto de Ciências Biológicas, Universidade Federal de Minas Gerais, 31270-901 Belo Horizonte, MG, Brazil

## Abstract

*Leishmania amazonensis* is the etiologic agent of the cutaneous and diffuse leishmaniasis often associated with drug resistance. Lapachol [2-hydroxy-3-(3′-methyl-2-butenyl)-1,4-naphthoquinone] displays a wide range of antimicrobial properties against many pathogens. In this study, using the classic microscopic *in vitro* model, we have analyzed the effects of a series of lapachol and chlorides complexes with antimony (V), bismuth (V), and tin (IV) against *L. amazonensis*. All seven compounds exhibited antileishmanial activity, but most of the antimony (V) and bismuth (V) complexes were toxic against human HepG2 cells and murine macrophages. The best IC_50_ values (0.17 ± 0.03 and 0.10 ± 0.11 *μ*g/mL) were observed for Tin (IV) complexes (**3**) [(Lp)(Ph_3_Sn)] and (**6**) (Ph_3_SnCl_2_), respectively. Their selective indexes (SIs) were 70.65 and 120.35 for HepG2 cells, respectively. However, while analyzing murine macrophages, the SI decreased. Those compounds were moderately toxic for HepG2 cells and toxic for murine macrophages, still underlying the need of chemical modification in this class of compounds.

## 1. Introduction

*Leishmania amazonensis*, a New World species, has been identified as a dermotropic species often associated with drug resistance [1]. Current antileishmanial therapies are toxic to human and some simply fail [2, 3]. In the Americas, for over six decades, parenteral administrations of pentavalent antimonials (Sb-V), sodium stibogluconate (Pentostam), and meglumine antimoniate (Glucantime) have been used for treating leishmaniasis. In places where resistance to antimonials is common, such as India, other chemotherapeutic treatments include amphotericin B and pentamidine [2, 4]. Therefore, the absence of a low toxic and safe oral drug still underlines the need for new antileishmanial compounds.

**Figure 1:**
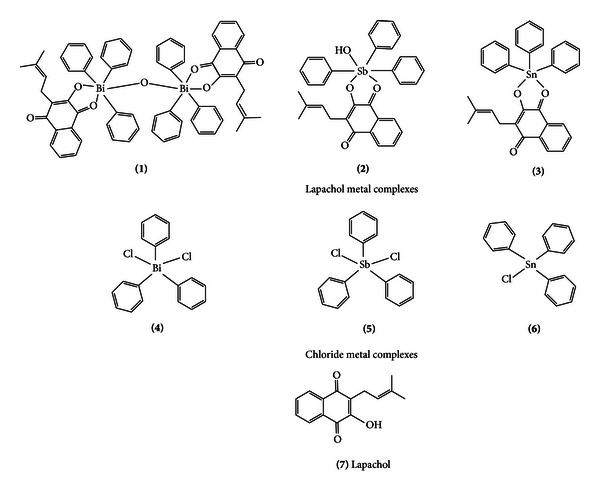
Structures of lapachol metal (Bi, Sb, and Sn) complexes (**1**–**3**) and chloride metal (Bi, Sb, and Sn) compounds (**4**–**6**) and lapachol (**7**). Legend: Bi = bismuth, Sb = antimony, and Sn = tin.

Lapachol, [2-hydroxy-3-(3′-methyl-2-butenyl)-1,4-naphthoquinone] (Figure 1) is a natural compound extracted from the core of Bignoniaceae trees. In *Leishmania*, lapachol analogues, derivatives, and complexes have been tested by several groups. Lapachol, isolapachol, and some of their derivatives were active *in vitro* and *in vivo* against *Leishmania braziliensis* and *L. amazonensis*, respectively [5]. Bismuth (III), antimony (V), and tin (IV) complexes were active against *Helicobacter pylori*, *Leishmania major*, and *Leishmania donovani*, respectively [6–8].

The design of bifunctional metal complex, where both the ligand and the metal exert pharmacological activity, represents a promising strategy for achieving more effective and selective drugs. In the present study, lapachol was coupled with three different metals: triphenyltin (IV), triphenylbismuth (V), and triphenylantimony (V). We have tested the *in vitro* activity and cytotoxicity of synthesized antimony (V), bismuth (V), and tin (IV) lapachol and chloride complexes against intracellular *L. amazonensis*, HepG2 cells, and murine macrophages.

## 2. Materials and Methods

### 2.1. Synthesis of the Lapachol Metal Complexes and Tested Metal Chlorides

The (Lp)(Ph_3_Bi)O_0.5_  (1) and (Lp)(Ph_3_Sb)OH (**2**) complexes were synthesized by following the procedure described by [9]. To prepare (Lp)(Ph_3_Sn) (**3**) the same procedure was used. Triethylamine (70 *μ*L) was added to a mixture of lapachol (0.121 g, 0.5 mmol) and triphenyltin (IV) chloride (193 mg, 0.5 mmol) in chloroform (20 mL). The resulting mixture was stirred for 4 h at room temperature. Removal of the solvent under vacuum yielded a solid material. The material was subsequently dissolved in acetone and precipitated in water. The triethylammonium hydrochloride formed during the reaction was dissolved and removed by water. Elemental analyses were carried out using a Perkin-Elmer 240 Elemental Analyzer. Atomic absorption analyses of bismuth, antimony and tin contents were carried out on a Hitachi Atomic Absorption Spectrophotometer (Model 8200).

The following equations can be proposed to illustrate the formation of (Lp)(Ph_3_Sb)OH complex as follows:

(1) LpH+Et3NH→THFLp−+[Et3NH]+Lp−+Ph3SbCl2+[Et3NH]+  →THFLpSbPh3Cl+[Et3NH]ClLpSbPh3Cl+[Et3NH]Cl+H2O  →LpSbPh3OH+[Et3NH]+HCl

The same process can be proposed for all complexes. The yields, melting points, and elemental analyses of the compounds prepared are given in Table 1.

Triphenylbismuth dichloride (**4**), triphenylantimony dichloride (**5**), triphenyltin chloride (**6**), and lapachol (**7**) were obtained from Aldrich. Triethylamine was obtained from Sigma. The predicted structures of all tested compounds are shown in Figure 1.

### 2.2. Parasites

The World Health Organization (WHO) reference strain* L. amazonensis* (IFLA/BR/1967/PH8) was used and typed as previously described [10]. Promastigote forms were grown at 25°C in M199 medium (Sigma) supplemented with 10% heat-inactivated fetal calf serum (Cultilab), 40 mmol/L HEPES (Amersham), 0.1 mmol/L adenine (Sigma), 0.0005% hemin (Sigma), 0.0002% biotin (Sigma), 50 units/mL penicillin, and 50 mg/mL streptomycin (Invitrogen) [11].

### 2.3. *In Vitro* Classic Microscopic Tests

Animals were kept in the Animal Facility of the Centro de Pesquisas René Rachou/FIOCRUZ in strict accordance to the Guide for the Care and Use of Experimental Animals [12]. The procedures were approved by the Internal Ethics Committee in Animal Experimentation (CEUA) of Fundação Oswaldo Cruz (FIOCRUZ), Brazil (Protocol L-042/08). Mice were euthanized with CO_2_ in an induction chamber prior to macrophage removal. Balb/c mice were injected intraperitoneally with 2 mL of 3% sodium thioglycollate medium. After 72 h, peritoneal macrophages were removed by washing with cold RPMI 1640 medium and enriched by adherence to round glass coverslips (13 mm) placed in a 4-well culture plate. Cells (2 × 10^5^ cells/well) were cultured (37°C, 5% CO_2_, 18 h) in RPMI supplemented with 10% heat-inactivated FBS (fetal bovine serum) prior to infection with parasites. Macrophages were exposed to stationary phase promastigotes (2 × 10^6^/well) at a final ratio of 1 : 10. The plates were incubated at 37°C, 5% CO_2,_ for 5 h in BOD to allow internalization of parasites [13]. Then, the medium was removed for the remaining noninternalized parasites. Negative control included only infected macrophages and medium. Incubations were tested in duplicate in two independent experiments [14, 15]. The substances were serial diluted with RPMI 1640 medium supplemented with 10% FBS at five different concentrations (50 → 3.1 *μ*g/mL). For compounds (**3**) and (**6**), the dilution was 10 → 0.016 *μ*g/mL. Amphotericin B was used as reference drug. Infected macrophages were exposed daily to the compounds for 3 consecutive days. After this period, coverslips were collected, stained with Panoptic (Laborclin), and subsequently mounted with Entellan (Merck) on glass slides.

### 2.4. Cytotoxicity Tests

The cell lineage HepG2 A16 was derived from a human hepatocellular carcinoma cell line HepG2 (ATCC HB-8065) and obtained from America Type Culture Collection line (ATCC) [16]. Balb/c murine peritoneal macrophages were obtained as described above. Cytotoxicity was determined using the MTT method (3-(4,5-dimethylthiazol-2-yl)-2,5-diphenyl tetrazolium bromide) (Sigma). HepG2 cells were kept in RPMI medium supplemented with 10% FBS, and confluent monolayers were trypsinized, washed in RPMI, and transferred to 96-well microtiter plates (4 × 10^4^ cells/well) for 16–18 h. Murine macrophages were used in the concentration 2 × 10^5^ cells/well in 96-well microtiter plates. The compounds were serial diluted in different concentrations (10 → 0.16 mg/mL). In both tests, the medium was removed, and the compounds were incubated for 24 h (37°C, 5% CO_2_). Colorimetric reaction was developed following the incubation with MTT (37°C, 5% CO_2_, 4 h) and addition of acidified isopropanol [17]. The reaction was read spectrophotometrically (Spectramax M5, Molecular Devices, San Francisco, CA) with a 570 nm filter and a background of 670 nm. Incubations were tested in triplicate in two independent experiments. The minimum dose that killed 50% of the cells (MLD_50_) was determined [18], and the values were plotted to generate dose-response curves using Microcal Origin Software (Northampton, MA, USA) [15, 19]. The selective indexes (SIs) of compounds were calculated using the MLD_50_/IC_50_ ratios to HepG2 and peritoneal macrophages [20, 21].

## 3. Results

**Figure 2:**
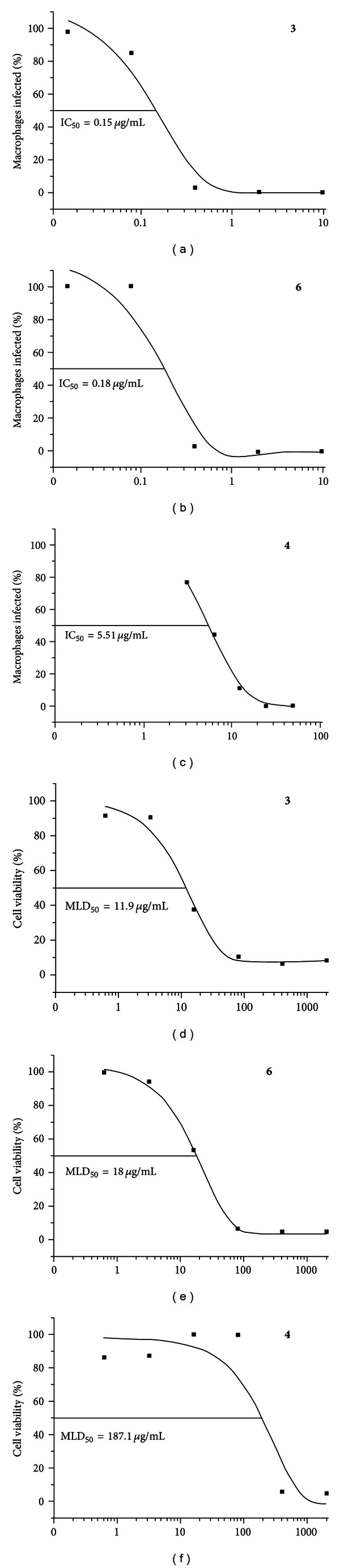
*In vitro* antileishmanial activity of compounds (**3**), (**4**), and (**6**) against intracellular *L. amazonensis* ((a), (b), and (c)) and cytotoxicity against hepatoma HepG2 cell ((d), (e), and (f)). Curves were obtained using Microcal Origin Software. IC_50_ = half-maximal inhibitory response; MLD_50_ = the minimum lethal dose. Figures are a representation of one experiment.

The *in vitro* classic microscopic test enables direct counting to determine the percentage of infected cells and/or the number of amastigotes [22]. Here, the IC_50_ values were calculated based on the percentage of infected macrophages [15]. The *in vitro* antileishmanial activities, cytotoxicity and selective indexes (SIs) of lapachol metal complexes and chlorides (**1**–**6**), lapachol (**7**) and amphotericin B are shown in Table 2. Lapachol and compounds (1), (**2**), and (**5**) were considered inactive (IC_50_ > 10 *μ*g/mL) and toxic (SI < 20) for HepG2 cells and macrophages [20, 21]. The tin (IV) lapachol complex (**3**) and chloride (**6**) were active against intracellular amastigote forms of *L. amazonensis* (Figures 2(a) and 2(b)) and less toxic for HepG2 cells (SIs ranging from 70.65 to 120.35) (Figures 2(d) and 2(e)) (Table 2). One triphenyl bismuth chloride (**4**) (Figure 2(c)) was also active and a little more toxic for HepG2 cells (Figure 2(f)) than (**3**) (Figure 2(d)) and (**6**) (Figure 2(e)) (SI = 34.03). All compounds were toxic for murine macrophages (SI < 20). Amphotericin B, an antileishmanial reference drug, exhibited an IC_50_ value approximately fourfold higher than (**3**) and (**6**) (0.73 ± 0.60 *μ*g/mL) (Table 2).

## 4. Discussion

Leishmaniases are considered by the WHO as one of the major six important infectious diseases worldwide. Over the past years, the absence of research and development for new medicines targeting diseases affecting people in developing countries has become a global concern [23]. Currently, the development of new drugs, combinations, or protocols against tropical and neglected diseases is of great importance in public health [24–27]. However, side effects, treatment failure due to parasite resistance, HIV coinfection, and intravenous administration are the major concerns hindering leishmaniasis chemotherapy [2, 3].

Lapachol derivatives and complexes have exhibited antitumor, anti-inflammatory, antiangiogenic, analgesic, and antimicrobial properties [6, 28–32]. Lapachol and some of its analogues demonstrated activity *in vitro* against *L. braziliensis *and *L. amazonensis* [5]. The use of metal complexes against *Leishmania *may represent a potential alternative against the disease since antimony-based regimens tend to be very toxic. In this context, we have explored the use of lapachol and chloride metal complexes with antimony (V), bismuth (V), and tin (IV)] against *L. amazonensis*.

In contrast to data from previous studies, lapachol (**7**) did not exhibit significant antileishmanial activity against *L. amazonensis *(15.48 ± 5.23 versus 5.2 ± 0.70 *μ*g/mL) [5]. This IC_50_ value is close to that observed for *L. braziliensis *(11.9 ± 6.9 *μ*g/mL). This discrepancy could be attributed to the strain of *L. amazonensis *used (MHOM/BR/77/LTB0016) and experimental conditions. The highest antiproliferative activity against intracellular *L. amazonensis* was observed for tin (IV) lapachol and chloride complexes (**3**) and (**6**) (Figures 2(a) and 2(b)) and one bismuth (V) chloride compound (**4**) (Figure 2(c)). More importantly, compounds (**3**) and (**6**) were more active than amphotericin B and less toxic among all substances tested while using HepG2 cells (SIs of 70.65 and 120.35, resp.). Interestingly, the resulting compound of lapachol and tin (IV) showed a marked decrease in metal toxicity than lapachol alone (SIs of 70.65 versus 13.03, resp.). One of the possibilities that could justify such phenomenon could be due to an increase in the lipophilicity of the lapachol-complexed molecule. Another hypothesis is that lapachol complexation could affect the REDOX potential of the compound, thus, consequently changing its activity. Consistent with this idea, the mechanisms underlying those activities are related to the generation of reactive oxygen radicals (ROSs) induced by the bioreduction of its quinonoid nucleus through specific enzymes and oxygen [33–35]. ROS mechanisms induced by lapachol have been implicated in the chemotherapeutic activities against many protozoa such as *Trypanosoma cruzi *[30] and also tumor cells [31]. Similarly, among all metal chloride substances, the triphenyl tin (IV) chloride compound exhibited lower toxicity compared to bismuth (V) and antimony (V) chloride ones. Finally, compound (**4**) exhibited moderate toxicity (SI = 34.03) with an IC_50_ value 7-fold higher than amphotericin B. However, when cytotoxicity was tested against murine macrophages, the host cells for *Leishmania*, all compounds were toxic. Those data indicate the need of chemical modifications in this class of compounds in the search of novel antileishmanial molecules.

## 5. Conclusions

Lapachol and a series of six lapachol and chloride metal complexes have been evaluated for their *in vitro* activity against intracellular amastigote forms of *L. amazonensis*. The tin (IV) lapachol and chloride complexes (**3** and **6**) exhibited higher antileishmanial activity compared to amphotericin B. The triphenyl bismuth (V) compound (**4**) also exhibited antileishmanial activity with moderate cytotoxicity. Lapachol compounds with bismuth (V) and tin (IV) were less toxic when compared with lapachol alone for HepG2 cells. In conclusion, tin, and in a less extent, bismuth complexes were moderately toxic for HepG2 cells and toxic for murine macrophages.

## Figures and Tables

**Table 1:** Yields and elemental analyses of the compounds.

Compound	Yield (%)	M.p. (°C)^a^	C found (calc.) (%)	H found (calc.) (%)	Metal found (calc.) (%)	Formula for calc.
(1)	79	126–129	57.31 (57.40)	4.09 (4.23)	29.03 (29.98)	(Lp)(Ph_3_Bi)O_0.5_
(2)	76	154–156	65.30 (64.82)	4.52 (4.78)	20.64 (19.19)	(Lp)(Ph_3_Sb)OH
(3)	79	107–109	66.41 (67.03)	4.40 (4.77)	21.74 (20.07)	(Lp)(Ph_3_Sn)

^a^M.p.: melting point.

**Table 2:** Antileishmanial activity, cytotoxicity, and selective indexes of tested compounds for HepG2 cells and murine macrophages.

Compound	Formula	IC_50_^a^	HepG2		Macrophages	
			MLD_50_^b^	SI^c^	MLD_50_^b^	SI^c^
(1)	(Lp)(Ph_3_Bi)O_0.5_	29.05 ± 18.45	58.38 ± 8.47	2.01	32.4 ± 8.20	1.11
(2)	(Lp)(Ph_3_Sb)OH	18.27 ± 5.58	325.22 ± 89.40	17.81	130.65 ± 40.52	7.15
(3)	(Lp)(Ph_3_Sn)	0.17 ± 0.03	12.01 ± 0.17	70.65	1.6 ± 0.57	9.41
(4)	Ph_3_BiCl_2_	5.40 ± 0.16	183.75 ± 4.77	34.03	25.15 ± 0.49	4.67
(5)	Ph_3_SbCl_2_	11.61 ± 7.85	157.46 ± 37.13	13.56	30.75 ± 6.01	2.65
(6)	Ph_3_SnCl_2_	0.10 ± 0.11	12.04 ± 8.42	120.35	0.73 ± 0.13	7.30
(7)	Lp^d^	15.48 ± 5.23	201.77 ± 5.32	13.03	184.65 ± 6.58	11.92
Amphotericin B		0.73 ± 0.60	644.59 ± 126.57	883.00	179.95 ± 8.84	246.51

^a^IC_50_: the inhibitory concentration that killed 50% of the *L. amazonensis* in *μ*g/mL.

^b^MLD_50_: the minimum lethal dose that killed 50% of the cells in *μ*g/mL.

^c^SI: selective index, calculated based on the MLD_50_/IC_50_ ratios.

^d^Lp: Lapachol.
